# Supplementary material for: A benchmarking framework for comparative evaluation of low-complexity region detection tools in the human proteome
Source: Sci Rep. 2026 Apr 15;16:17498. doi: 10.1038/s41598-026-48432-3 (PMC13237088; doi:10.1038/s41598-026-48432-3)
Supplement: Supplementary file 1 — Supplementary Material 1 [file 41598_2026_48432_MOESM1_ESM.pdf]

SUPPLEMENTAL FIGURES WITH LEGENDS

A Benchmarking Framework for Comparative Evaluation of Low-Complexity Region Detection Tools in the Human Proteome

Anirjit Chatterjee[1], Nagarjun Vijay[1]

Computational Evolutionary Genomics Lab, Department of Biological Sciences, IISER Bhopal, Bhauri, Madhya Pradesh, India

Author for Correspondence: Anirjit Chatterjee, anirjit23@iiserb.ac.in, Nagarjun Vijay, nagarjun@iiserb.ac.in

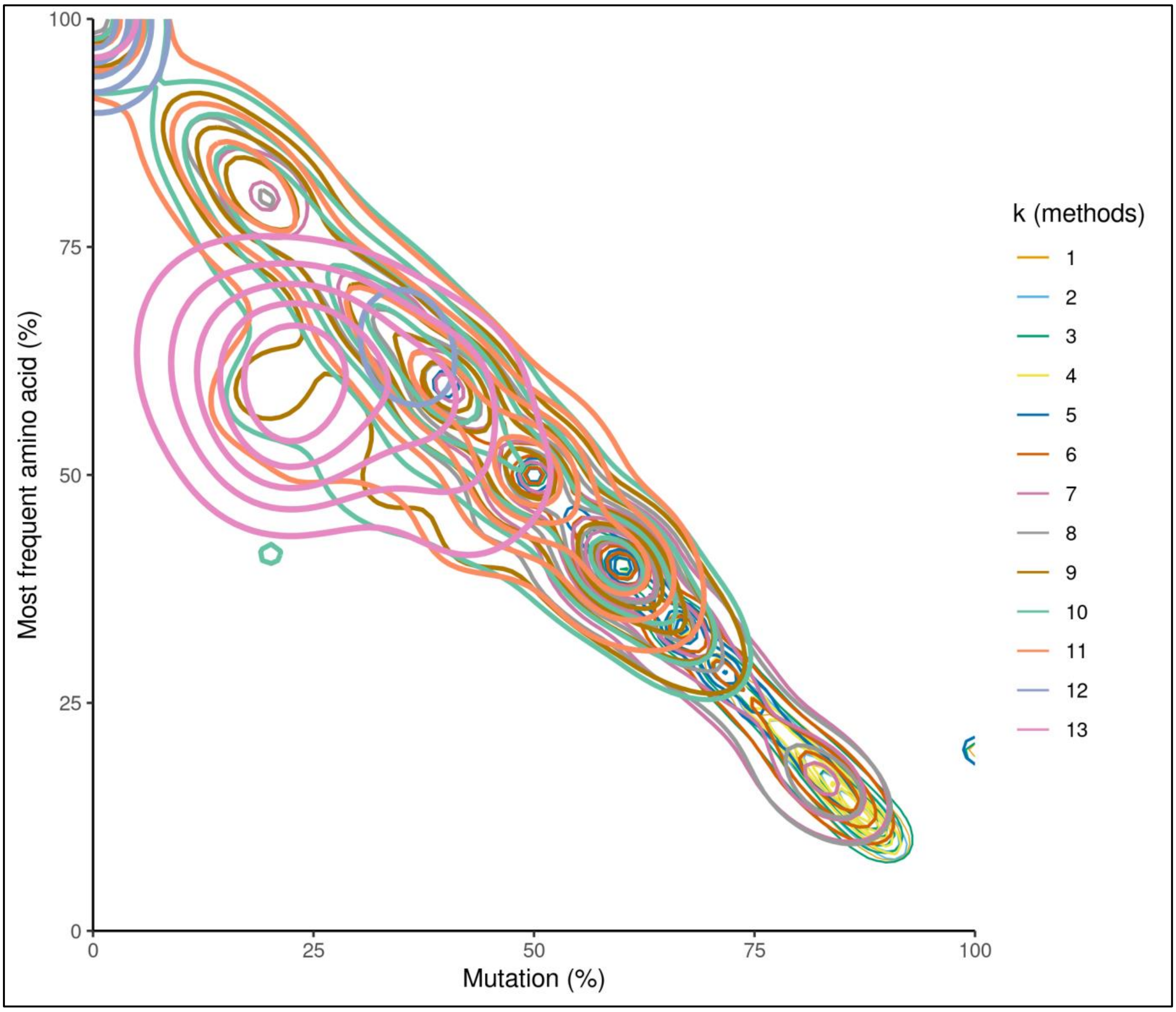

Supplementary Figure 1. Shared LC manifold sharpened by consensus. Kernel density contours of LC regions in mutation–composition space, colored by consensus tier (k). Contours represent increasing kernel density of LC regions.

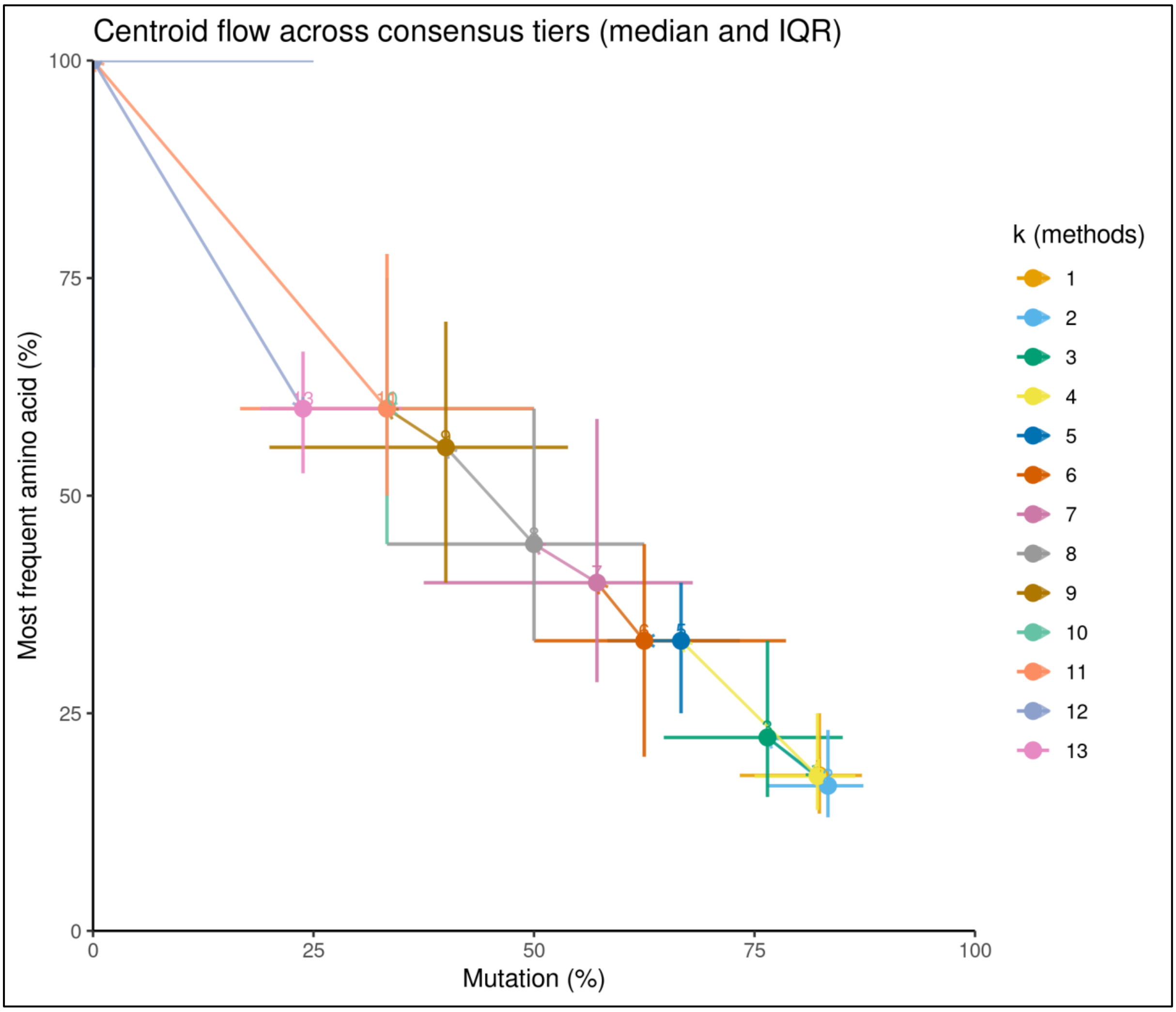

Supplementary Figure 2. **Consensus progression follows a directional complexity gradient (towards higher compositional dominance and lower mutation).** Centroid flow plot showing median positions and interquartile ranges of mutation percentage and most frequent amino acid fraction for each consensus tier (k).

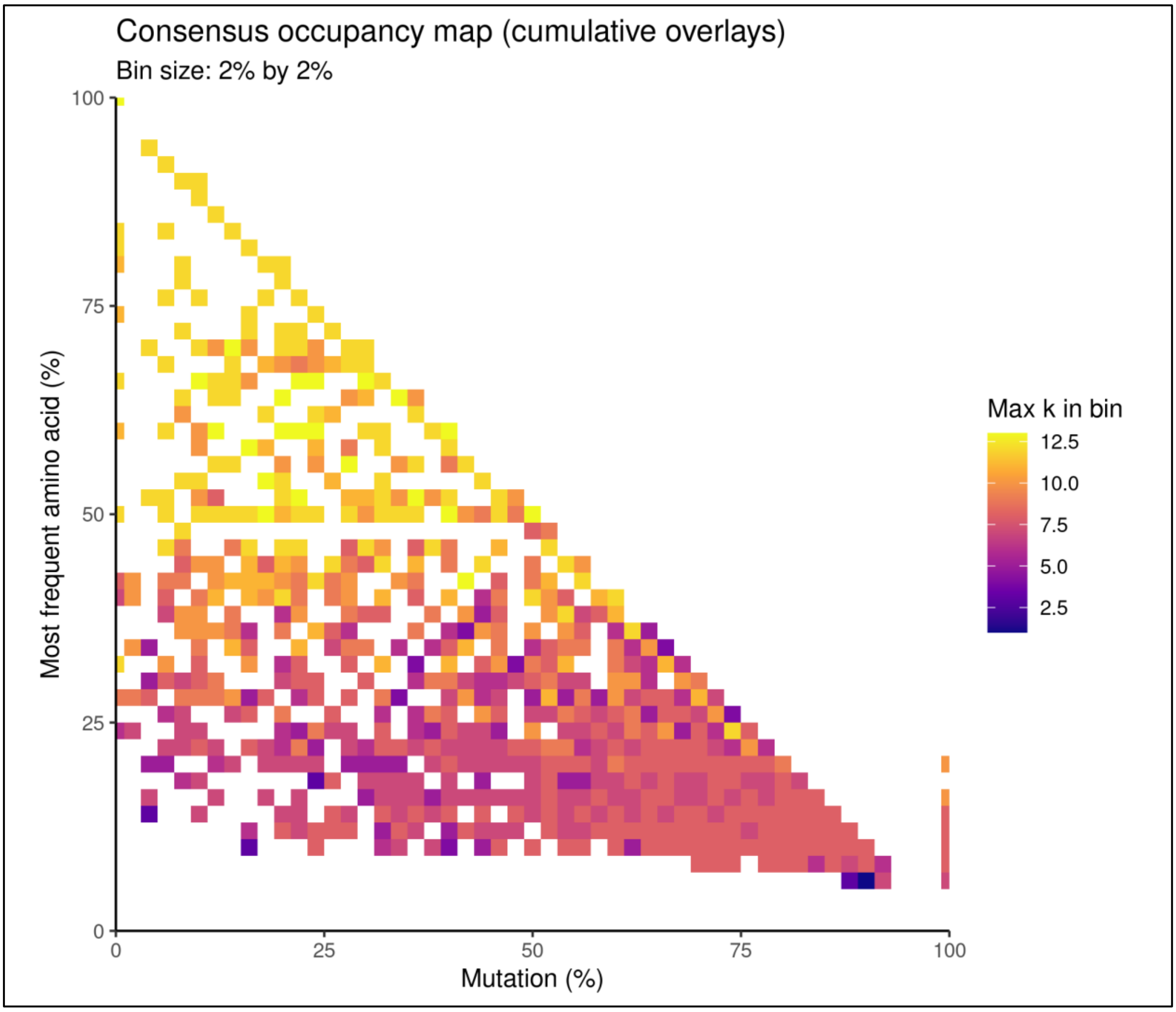

Supplementary Figure 3. Maximal method agreement concentrates in the intermediate LC space. Consensus occupancy heatmap showing the maximum number of overlapping methods (k) per 2% × 2% bin in LC space.

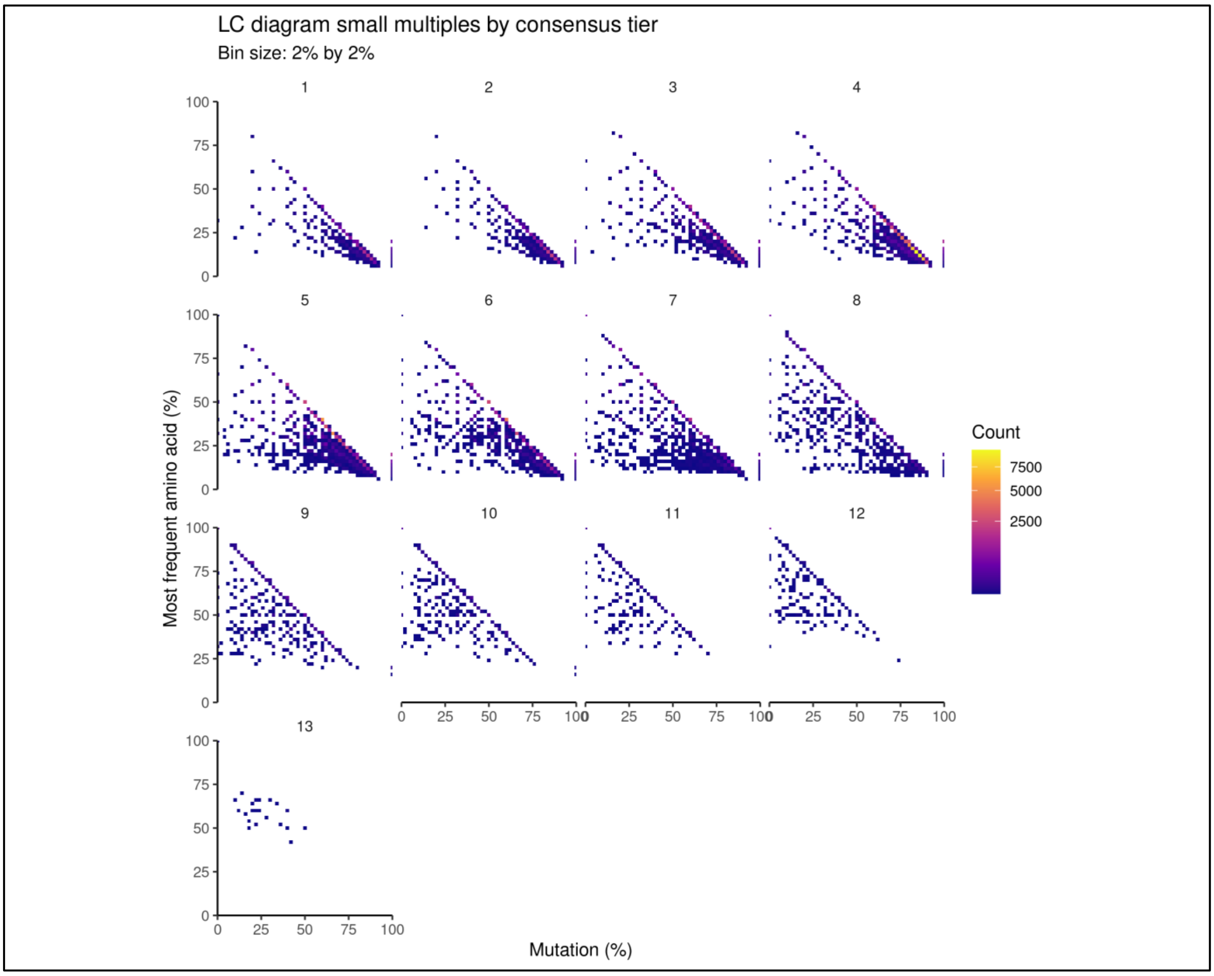

Supplementary Figure 4. Increasing consensus is associated with regions showing higher mutation percentages. LC diagram small multiples stratified by consensus tier (k = number of methods). Each panel shows binned occupancy (2% × 2%) of regions in mutation percentage versus the most frequent amino acid fraction. Colour denotes bin counts.

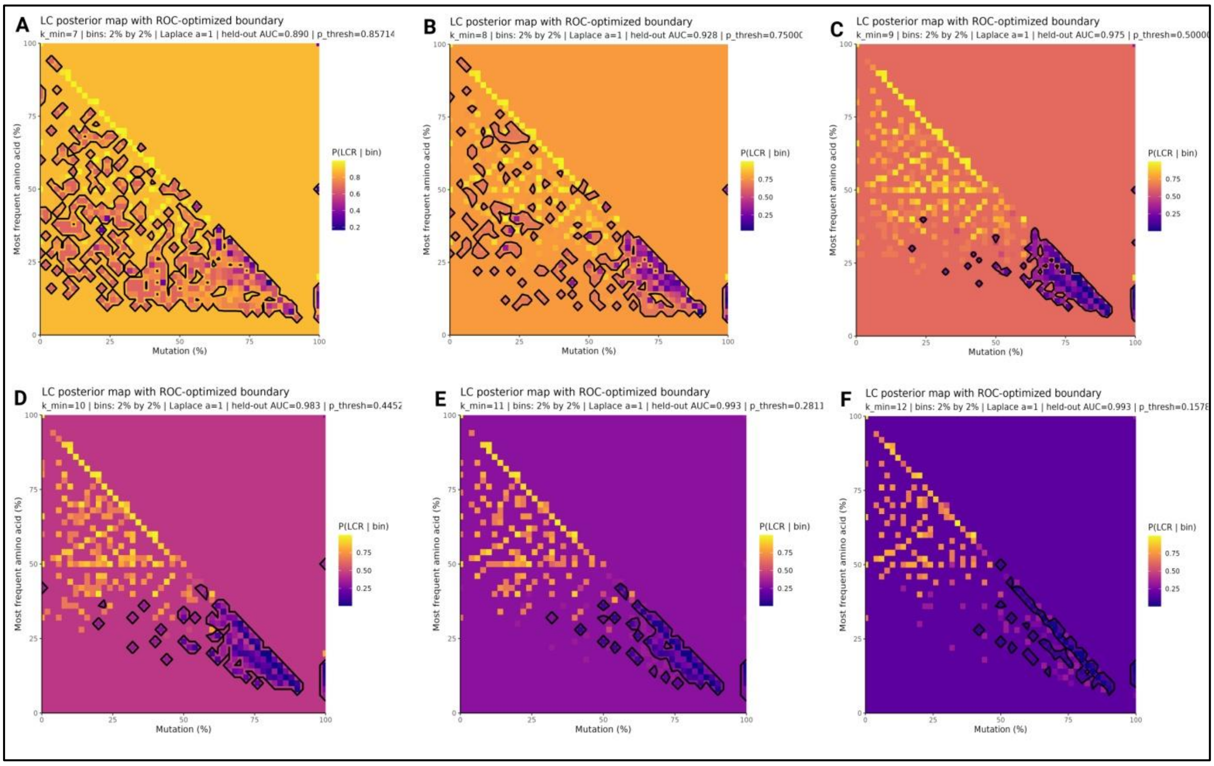

Supplementary Figure 5. UniProt-derived LC posterior maps and ROC-optimised boundaries. (A–F) LC posterior probability maps inferred from UniProt low-complexity annotations for increasing minimum window sizes (k = 7–12) [k = number of methods]. Each panel displays P(LC | bin) across mutation percentage (x-axis) and most frequent amino acid percentage (y-axis), with warmer colours indicating higher LC probability. Black contours indicate ROC-optimised decision boundaries obtained from held-out validation data (actual coordinates of the IDRs and the missing residues).

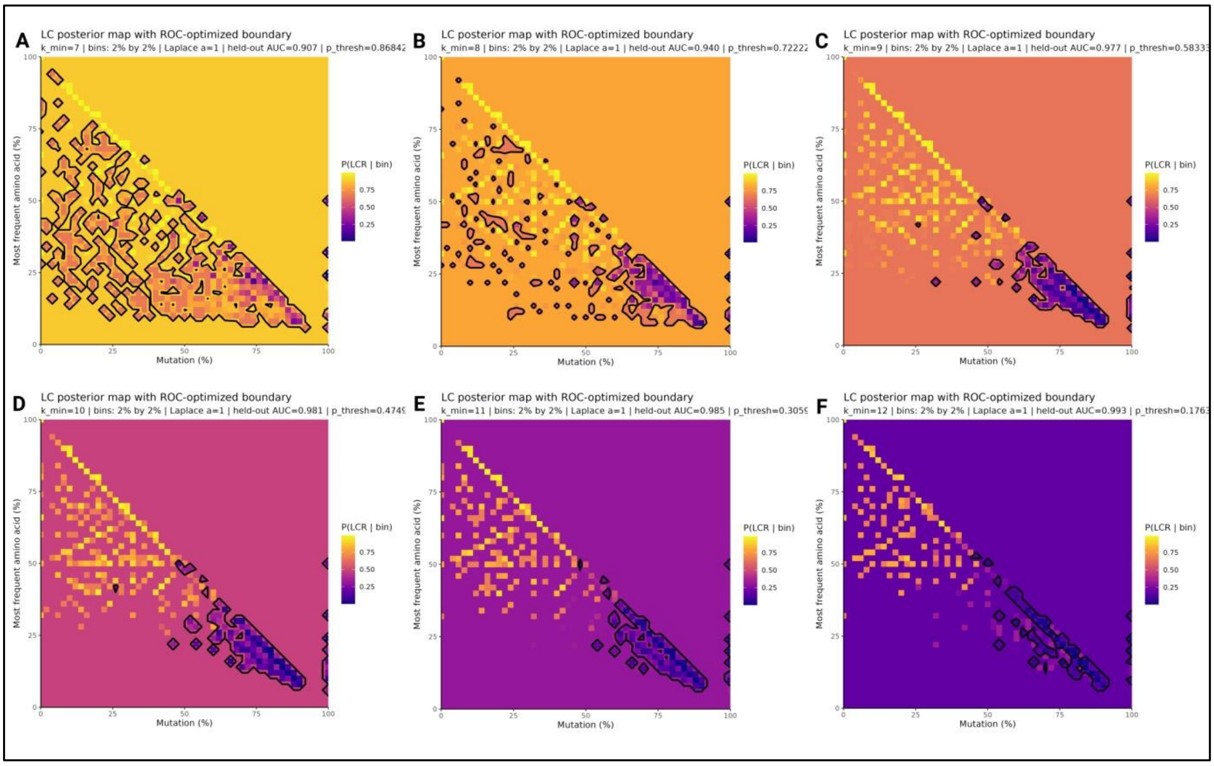

Supplementary Figure 6. DisProt-derived LC posterior maps and ROC-optimised boundaries. (A–F) LC posterior probability landscapes derived from DisProt-annotated intrinsically disordered regions across k = 7–12 [k = number of methods]. Heatmaps show P(LC | bin) as a function of mutation percentage and compositional dominance. ROC-optimised boundaries (black contours) are broader and more fragmented at lower k, reflecting higher entropy tolerance in disorder annotations.

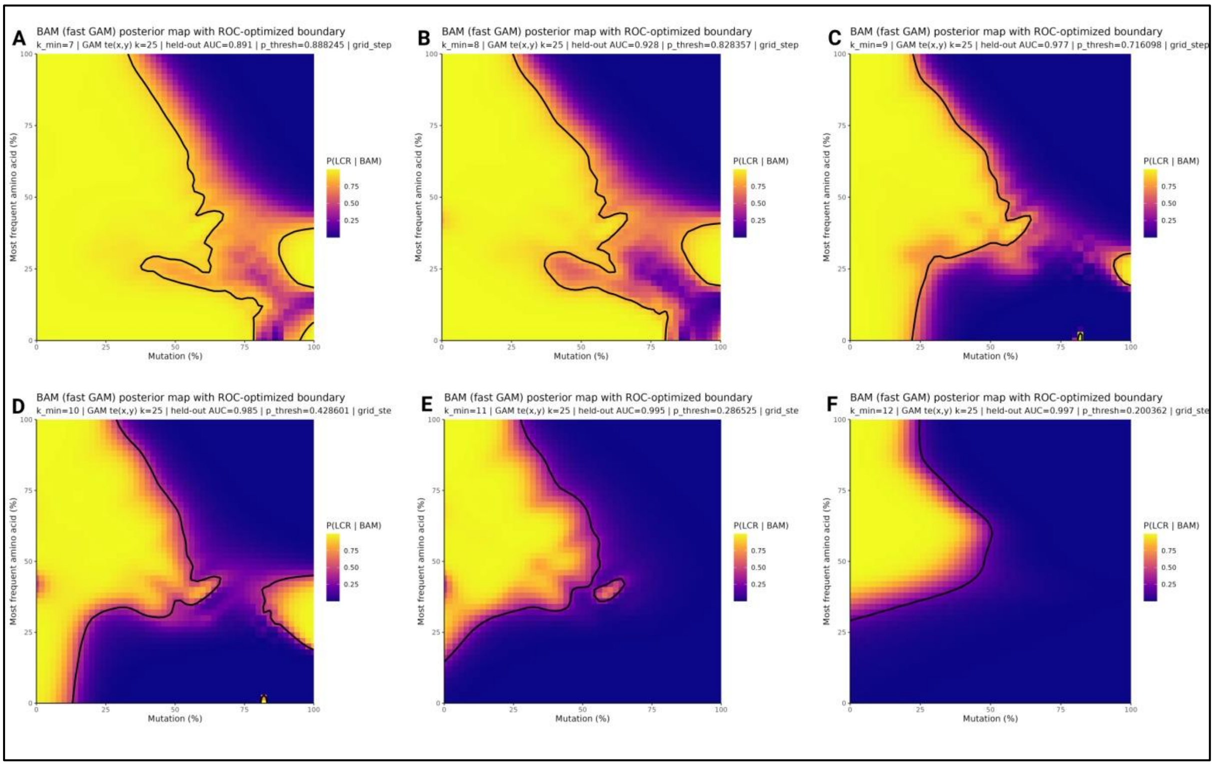

Supplementary Figure 7. BAM LC posterior maps using UniProt annotations. (A–F) Bayesian Additive Model (BAM) posterior probability surfaces trained on UniProt LC annotations for k = 7–12 [k = number of methods]. Smooth posterior gradients depict P(LC|BAM) across mutation and compositional axes. Black curves indicate ROC-optimised decision boundaries derived from the continuous posterior surface.

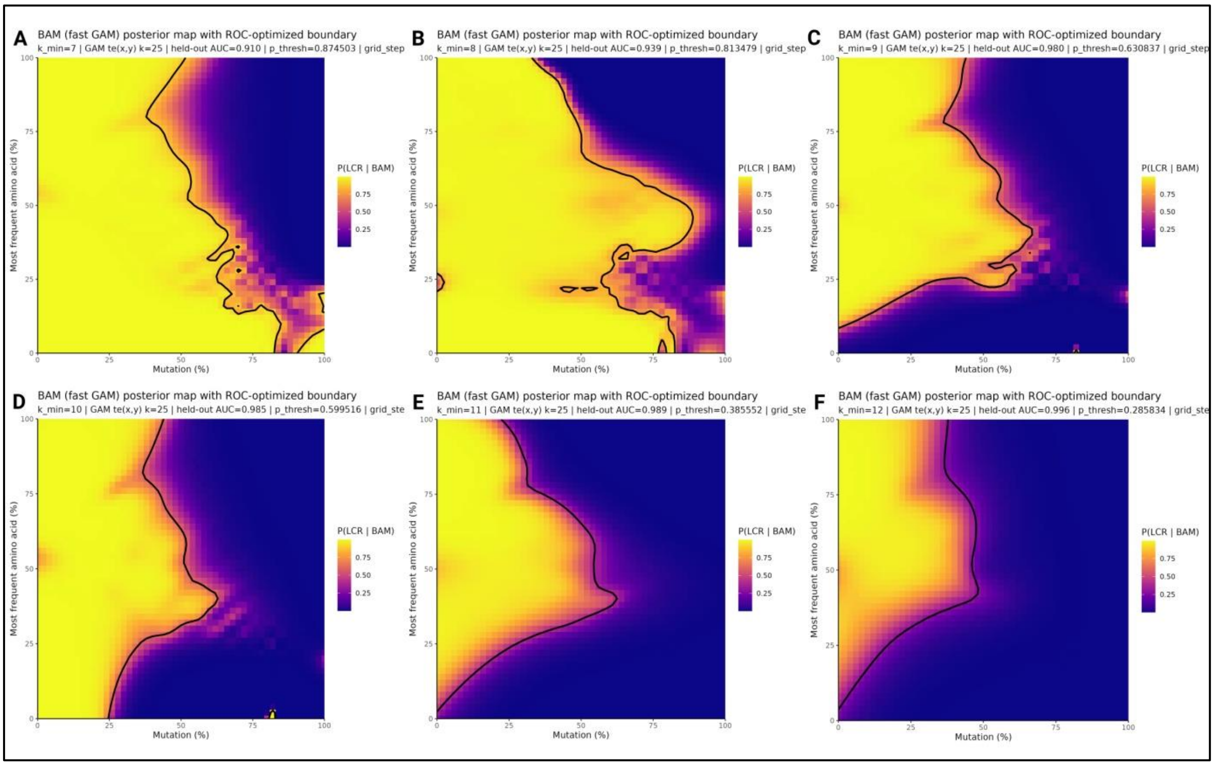

Supplementary Figure 8. BAM (fast GAM) LC posterior maps using DisProt annotations. (A–F) BAM-derived LC posterior probability maps trained on DisProt annotations across increasing k (7–12) [k = number of methods]. Posterior surfaces reveal expanded LC space at lower k, with smooth entropy-driven transitions across mutation and compositional dimensions. ROC-optimised boundaries (black curves) remain broader than UniProt-based counterparts but contract and regularise with increasing k.

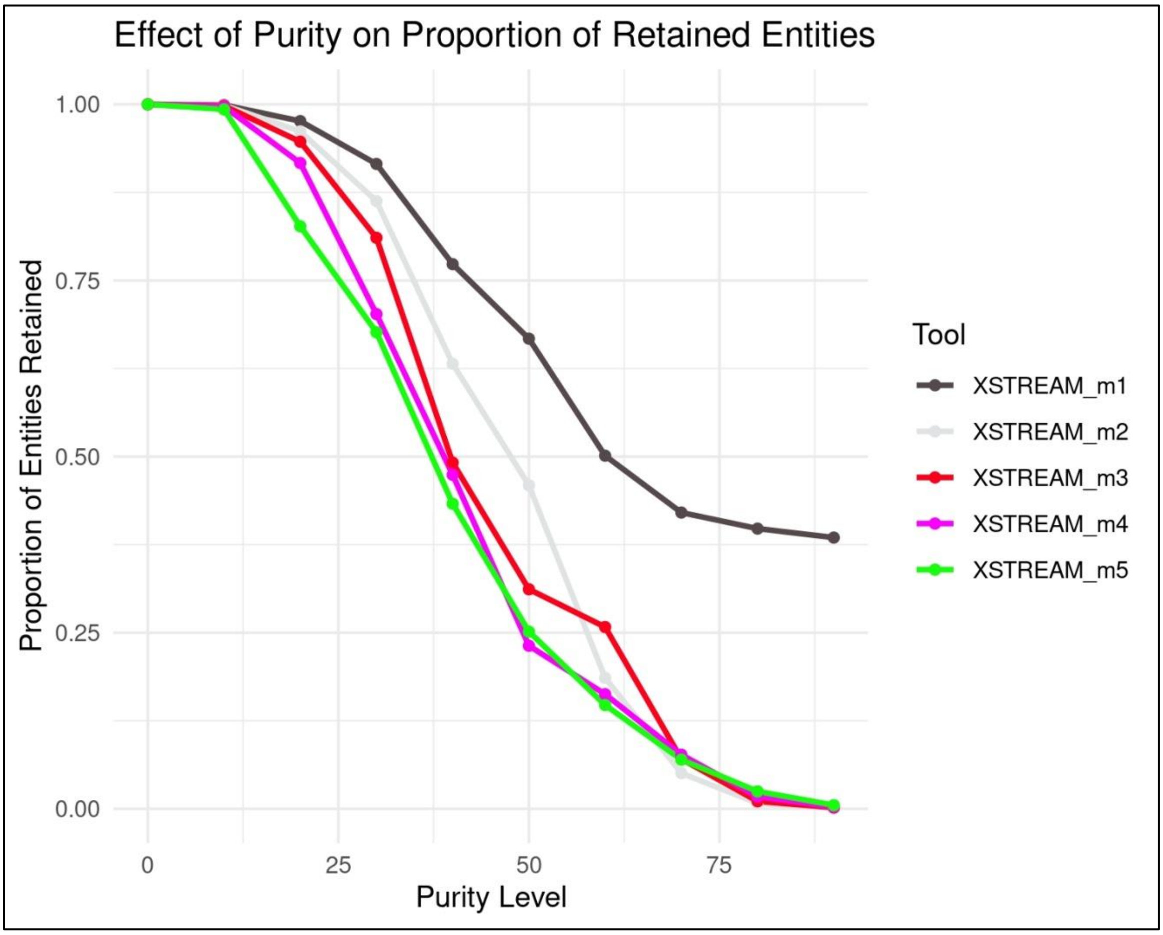

Supplementary Figure 9. Proportion of LCRs retained at increasing purity thresholds by XSTREAM (at various -m parameters). This figure shows the variation in LCR purity by XSTREAM (at various -m parameters). The x-axis represents the purity level, defined as the percentage of the most dominant amino acid within each LCR, while the y-axis denotes the proportion of total LCRs retained at or above each purity threshold. Each line corresponds to a different -m value, as indicated by the colour-coded legend. Steeper declines indicate detection of fewer highly pure (compositionally biased) regions, whereas gradual slopes reflect identification of more homogeneous LCRs. This comparison emphasises that XSTREAM varies in its sensitivity to amino acid repetitiveness at various -m values.
